# Therapeutic Effects of Lipid Lowering Medications on Myocardial Blood Flow, Inflammation, and Sympathetic Nerve Activity Using Nuclear Techniques

**DOI:** 10.1007/s11886-022-01792-4

**Published:** 2022-10-13

**Authors:** Takahiro Higuchi, Sebastian E. Serfling, Steven P. Rowe, Rudolf A. Werner

**Affiliations:** 1 Department of Nuclear Medicine, University Hospital Würzburg, Oberdürrbacherstr. 6, 97080 Würzburg, Germany; 2 Faculty of Medicine, Dentistry and Pharmaceutical Sciences, Okayama University, Okayama, Japan; 3 The Russell H Morgan Department of Radiology and Radiological Sciences, Johns Hopkins School of Medicine, Baltimore, MD USA

**Keywords:** Sympathetic nervous system, Cardiac nerve, MIBG, Inflammation, Blood flow, Statin

## Abstract

**Purpose of Review:**

Statins are routinely applied in patients with coronary artery disease, as they allow significantly to reduce blood cholesterol levels. Although those drugs are endorsed by current guidelines and prescribed routinely, a substantial portion of patients are still statin-intolerant and image-piloted strategies may then be helpful to identify patients that need further intensified treatment, e.g., to initiate treatment with proprotein convertase subtilisin / kexin type 9 inhibitors (PCSK9i). In addition, it has also been advocated that statins exhibit nonlipid, cardio-protective effects including improved cardiac nerve integrity, blood flow, and anti-inflammatory effects in congestive heart failure (HF) patients.

**Recent Findings:**

In subjects after myocardial infarction treated with statins, ^123I^I-metaiodobenzylguanidine (MIBG) scintigraphy has already revealed enhanced cardiac nerve function relative to patients without statins. In addition, all of those aforementioned statin-targeted pathways in HF can be visualized and monitored using dedicated cardiac radiotracers, e.g., ^123^I-MIBG or ^18^F-AF78 (for cardiac nerve function), ^18^F-flurpiridaz (to determine coronary flow) or ^68^Ga-PentixaFor (to detect inflammation).

**Summary:**

Statins exhibit various cardio-beneficial effects, including improvement of cardiac nerve function, blood flow, and reduction of inflammation, which can all be imaged using dedicated nuclear cardiac radiotracers. This may allow for in vivo monitoring of statin-induced cardioprotection beyond lipid profiling in HF patients.

## Introduction

**Fig. 1:**
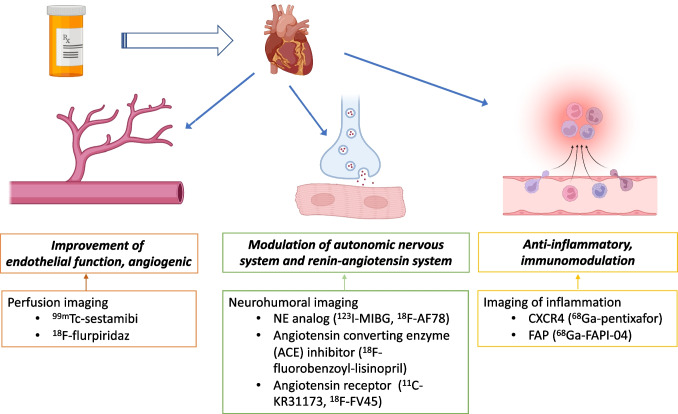
Beyond lowering cholesterol, statins may also exert beneficial effects, including improvement of endothelial function leading to improved blood flow, modulation of the sympathetic nervous and renin angiotensin aldosterone system, or immunomodulation. All of these pathophysiological pillars can be imaged using dedicated nuclear cardiac imaging techniques, including perfusion, neurohumoral, or inflammatory-targeted imaging and thus, those SPECT or PET radiotracers may allow to decipher the beneficial effects on every pathophysiological pathway. NE, norepinephrine; MIBG, metaiodobenzylguanidine; CXCR4, C-X-C motif chemokine receptor 4; FAPI, fibroblast activation inhibitor. Created with BioRender.com

Prescription of statins (3-hydroxy-3-methyl-glutaryl-coenzyme A reductase inhibitor [HMG-CoA]) causes a relevant decrease of hepatic cholesterol, thereby exhibiting substantial cardioprotective effects [1]. In this regard, a 1.0 mmol/L reduction of LDL cholesterol can prevent the incidence of major cardiovascular events, including stroke, myocardial infarction (MI), or revascularization by almost 10% [2]. Of note, in a recent meta-analysis pooling 170,000 patients of 26 prospective trials, the authors even conclude on an incremental value, leading to a potential risk reduction of 40–50% by LDL cholesterol reduction by 2–3 mmol/L [2]. Beyond exerting a beneficial effect on blood cholesterol level [1], mainly preclinical studies reported on an additional impact on cardiac autonomic function, e.g., by inhibition of angiotensin type II, also leading to improved left ventricular function [3]. Moreover, statins may also enhance epicardial perfusion in patients after MI scheduled for percutaneous coronary intervention (PCI) [4], further supporting the notion of a complex interplay between hepatic-derived reduction of blood cholesterol, cardiac nerve integrity, and myocardial blood flow [3, 4]. A recent study investigating 4622 patients who underwent cardiac magnetic resonance imaging reported that 17% received statins [5]. Those individuals had significantly less wall thickening relative to controls, which may be partially explained by statin-related reduction of oxidative stress [5], followed by decreased production of growth factors, but also to increased levels of nitric oxide synthase activity, leading to improved blood flow [6]. Further cardioprotective effects may also include anti-inflammatory components, as demonstrated by reduced C-reactive protein levels in individuals under regular statin prescription [7]. Of note, chronic heart failure is characterized by all of those pathophysiological pillars, thereby indicating that regular statin prescription may not only lower cholesterol blood levels but also exert relevant nonlipid therapeutic effects in patients affected with this disease [8]. In addition, those “off-target” effects of statins beyond lipid profiling may all be visualized and monitored by nuclear cardiac imaging techniques, including assessment of autonomous nerve function of the heart, myocardial blood flow, and pro-inflammatory activity (Fig. 1) [9]. As such, in the present review, we aimed to provide a brief overview of dedicated nuclear cardiac radiotracers applied in the context of lipid-lowering medication. By providing various clinical scenarios, we will also discuss how molecular imaging can be of relevance for the referring treating cardiologist when statins are prescribed.

## Clinical Benefit of Conducting Nuclear Imaging for Statin Prescription

Statins have already been proposed as part of a polypill, including multiple blood-lowering drugs and aspirin in patients post-MI [10]. In this regard, a fixed-dose concept is preferred over an individual dosage concept, thereby questioning the use of nuclear cardiac imaging techniques to determine the most appropriate drug dosage or to identify patients that benefit the most from treatment initiation [9]. In addition, side effects due to statins may not occur frequently, including myopathy or rhabdomyolysis [1]. Also summarized as statin-related muscle symptoms (SRMS) [1], it may be also debatable whether single photon emission computed tomography (SPECT) or positron emission tomography (PET) may be suitable to determine such high-risk patients if those SRMS are rare [9]. Nonetheless, there is a substantial portion of patients that do not respond to statins [11]. In those subjects with statin intolerance, the PCSK9i, which allows for the degradation of the hepatocyte LDL receptor, maybe a potential alternative to commonly prescribed HMG-CoA [12]. PCSK9i, however, is higher-priced, thereby indicating a need to identify patients that would benefit from such sophisticated lipid-lowering drugs, e.g., by risk stratification using nuclear cardiac imaging [13]. As such, an image-piloted strategy could also be integrated into the therapeutic algorithm of those subjects [9], e.g., to enhance the therapeutic efficacy of combination strategies using regular statins and PCSK9i [12]. Further clinical applications of image-guided nuclear cardiology imaging studies may also emerge once the beneficial effects of statins on blood flow, cardiac autonomic function, and anti-inflammation have been further determined.

## Molecular Imaging Targets in Patients under Statin

### Targeting Cardiac Nerve Integrity

First applied to symptomatic heart failure patients in a prospective setting, the “AdreView Myocardial Imaging for Risk Evaluation in Heart Failure (ADMIRE-HF)” trial has provided evidence that the catecholamine analog radiotracer ^123^I-metaiodobenzylguanidine (MIBG) may allow determining patients that are prone to NYHA functional class progression, life-threatening arrhythmias or myocardial death [14]. As a commonly derived metric, the heart-to-mediastinum ratio (HMR) allows for quantifying the amount of denervated myocardium, with reduced HMR being linked to an increasing event rate [14]. This SPECT radiopharmaceutical has been applied by Takahashi and coworkers to subjects with ST-segment elevation MI under statins and was then compared to a control group also diagnosed with infarction but not being treated with statins [15]. Individuals under statins showed higher HMR values on a delayed image protocol along with a lower washout rate, thereby indicating that ^123^I-MIBG allows to monitoring the beneficial effects of statin therapy in patients post-MI. Of note, those findings were accompanied by plasma procollagen type III amino terminal peptide (PIIINP), which is used to determine cardiac fibrosis. This biomarker and left ventricular performance were both improved in the statin group, further supporting the notion that other independent clinical parameters suggest a benefit of statin therapy similar to HMR [15]. Despite those interesting results in the context of SPECT, recent radiochemical developments led to the increasing use of PET agents for identifying areas of cardiac nerve injury [16]. Relative to SPECT, those PET agents may provide a higher spatiotemporal resolution, in particular when combined with F18 radiochemistry [17]. Those compounds include but are not limited to ^18^F-AF78, 18F-flubrobenguane, or 18F-DOPA [17, 18, 19••, 20]. If image-piloted strategies should be applied in the context of statins, e.g., to determine individuals that should be treated with PCSK9i and/or combination approaches, those more sophisticated PET radiotracers could then be used [13]. Beyond assessing cardiac nerve integrity, hypercholesterolemia is also tightly linked to the renin–angiotensin–aldosterone system, mainly due to the release of angiotensin II [21] and thereby supporting the notion that angiotensin-targeting PET radiotracers may also be applied in the context of statin, e.g., by using the angiotensin-converting-enzyme-targeting ^18^F-fluorobenzoyl-lisinopril [22] or AT-1 receptor ligand 11C-KR31173, which is also directed towards the angiotensin II subtype 1 receptor (AT1R) of the human heart [23]. The recently introduced PET radiotracer ^18^F-FV45 could also be selectively blocked by the clinical drug valsartan, and thus, this agent may further expand the current armamentarium of radiotracers targeting angiotensin [24].

### Targeting Cardiac Blood Flow

As alluded to earlier, a recent study including 4622 patients demonstrated that individuals under statin showed less wall thickening relative to controls [5]. As a possible explanation, statins may increase levels of nitric oxide synthase activity, improving blood flow [6]. Cerit and coworkers have provided further evidence of this hypothesis by enrolling 80 patients with a diagnosis of stable coronary artery disease scheduled for PCI [4]. Upon multiple logistic regression, statin pre-treatment along with high-sensitivity C-reactive protein (CRP) were independent predictors of post-interventional epicardial perfusion [4]. This is in line with an observation of Briguori et al. who also observed a potential beneficial impact of statins on coronary blood flow. In their study, the incidence of PCI-related peri-interventional MI was significantly reduced in statin-pretreated subjects. In this regard, the authors recommended a high-dose approach of 80 mg loading of atorvastatin one day prior to the elective intervention [25]. Taken together, statin may also play a crucial role in improving blood flow, and thus, myocardial perfusion imaging could play an important role to determine statin-related effects of improved coronary flow. As a workhorse in nuclear cardiology, the SPECT radiotracers 99mTc-sestamibi, -tetrofosmin, and Thallium 201 could then be applied in individuals under statin therapy and compared to controls not receiving a statin, e.g., in the setting of MI followed by reperfusion [9]. Again, PET may provide increased diagnostic accuracy and multiple blood flow-targeting radiotracers would be also available. Those include short-lived radiotracers such as 13N ammonia (half-life, 10 min), oxygen-15 water (2 min), or 82Rb (76 s), but also F18-labeled cardiac perfusion radiotracers, e.g., flurpiridaz [9, 17]. The latter radiotracer has significant advantages when compared to other radiotracers used for this purpose, as the longer half-life of 110 min would also allow conducting of early and delayed studies and high throughput in a busy PET center [17, 26]. In addition, it has the unique potential for injecting the radiotracer at peak exercise on the treadmill, which cannot be performed with the current FDA-approved PET radiotracers [27, 28].

### Targeting Inflammation

Beyond lipid-lowering effects, statins may also exert anti-inflammatory effects as another pathophysiological pillar of congestive heart failure. Among others, results of the CARE trial (Cholesterol and Recurrent Events) first reported a dual-targeting beneficial effect, including lower cholesterol levels and reduction of CRP [29]. Those findings were further corroborated in the JUPITER trial (Justification for the Use of Statins in Prevention: an Intervention Trial Evaluating Rosuvastatin), demonstrating that even in otherwise healthy subjects not diagnosed with hyperlipidemia but with elevated high-sensitive CRP, statins reduced the incidence of cardiovascular events, along with a substantial CRP decrease of 37% [30]. As such, statins may also exhibit a nonlipid, anti-inflammatory effect. Of note, inflammatory-targeted radiotracers are also penetrating the clinical arena. While 18F-fluorodeoxyglucose would be available at every PET center with access to a cyclotron (or via dispatch from a remote cyclotron facility) [31], challenging preparation protocols, including prolonged fasting, may hamper its widespread clinical adoption in the context of statin-related molecular imaging [32]. The C-X-C motif chemokine receptor 4 (CXCR4)-directed PET agent ^68^Ga-PentixaFor has already proven to provide predictive information in patients post-MI. Such a CXCR4-targeting, noninvasive local read-out of the inflammatory burden in the infarcted area could even better identify high-risk patients prone to later major cardiovascular events than established clinical markers, including creatine kinase, CRP, or leukocytes [33•]. Of note, ^68^Ga-PentixaFor does not require any particular patient preparation [34] and thus, patients with and without statins after myocardial infarction and guideline-compatible intervention could be easily compared using this radiotracer, thereby allowing to determine the anti-inflammatory effects of statins in vivo.

## Conclusions

Statins are routinely applied in patients with coronary artery disease, as they allow significantly to reduce blood cholesterol levels. Although statins are prescribed routinely, a substantial portion of patients are still statin-intolerant and image-piloted strategies, including molecular cardiac imaging, may then be helpful to identify patients that need further intensified treatment, e.g., initiation of PCSK9i. In addition, it has also been advocated that statins exhibit beneficial effects, including improved cardiac nerve integrity, blood flow, and anti-inflammatory effects in congestive heart failure. All of these pathophysiological pillars could be addressed using dedicated nuclear cardiac SPECT or PET radiopharmaceuticals, including ^123^I-MIBG, ^18^F-AF78, ^18^F-flurpiridaz, or ^68^Ga-PentixaFor. As such, those cardiac radiotracers may then allow for in vivo monitoring of statin-induced cardioprotection beyond lipid profiling in heart failure.
